# Synthesis and biological evaluation of NAS-21 and NAS-91 analogues as potential inhibitors of the mycobacterial FAS-II dehydratase enzyme Rv0636

**DOI:** 10.1099/mic.0.2008/017434-0

**Published:** 2008-07

**Authors:** Veemal Bhowruth, Alistair K. Brown, Gurdyal S. Besra

**Affiliations:** University of Birmingham, School of Biosciences, Edgbaston, Birmingham B15 2TT, UK

## Abstract

The identification of potential new anti-tubercular chemotherapeutics is paramount due to the recent emergence of extensively drug-resistant strains of *Mycobacterium tuberculosis* (XDR-TB). Libraries of NAS-21 and NAS-91 analogues were synthesized and evaluated for their whole-cell activity against *Mycobacterium bovis* BCG. NAS-21 analogues **1** and **2** demonstrated enhanced whole-cell activity in comparison to the parental compound, and an *M. bovis* BCG strain overexpressing the dehydratase enzyme Rv0636 was resistant to these analogues. NAS-91 analogues with *ortho*-modifications gave enhanced whole-cell activity. However, extension with biphenyl modifications compromised the whole-cell activities of both NAS-21 and NAS-91 analogues. Interestingly, both libraries demonstrated *in vitro* activity against fatty acid synthase II (FAS-II) but not FAS-I in cell-free extracts. In *in vitro* assays of FAS-II inhibition, NAS-21 analogues **4** and **5** had IC_50_ values of 28 and 19 μg ml^−1^, respectively, for the control *M. bovis* strain, and the *M. bovis* BCG strain overexpressing Rv0636 showed a marked increase in resistance. In contrast, NAS-91 analogues demonstrated moderate *in vitro* activity, although increased resistance was again observed in FAS-II activity assays with the Rv0636-overexpressing strain. Fatty acid methyl ester (FAME) and mycolic acid methyl ester (MAME) analysis of *M. bovis* BCG and the Rv0636-overexpressing strain revealed that the effect of the drug was relieved in the overexpressing strain, further implicating and potentially identifying Rv0636 as the target for these known FabZ dehydratase inhibitors. This study has identified candidates for further development as drug therapeutics against the mycobacterial FAS-II dehydratase enzyme.

## INTRODUCTION

The emergence of multi-drug resistant *Mycobacterium tuberculosis* (MDR-TB) (Kaye & Frieden, 1996) and the more recent identification of extensively drug-resistant *M. tuberculosis* (XDR-TB) (CDC, 2006) has highlighted the need for new TB drugs. Mycolic acids (C_60_–C_90_) are vital cell wall components of *M. tuberculosis* which form a lipid-rich permeability barrier. Currently, isoniazid represents the mainstay for chemotherapy against TB; it is known to target mycolic acid biosynthesis (Banerjee *et al.*, 1994). Mycolic acid biosynthesis involves both fatty acid synthase-I (FAS-I) and fatty acid synthase-II (FAS-II), with FAS-II being uniquely found in bacteria, plants and apicomplexan parasites, such as *Plasmodium* (Takayama *et al.*, 2005; Waller *et al.*, 2003). *M. tuberculosis* FAS-I catalyses *de novo* synthesis of intermediate-length (principally C_16_ and C_24_) fatty acids. FAS-II, however, is incapable of *de novo* fatty acid synthesis and accepts short-chain (C_16_) acyl-CoA primers from FAS-I via a condensation reaction carried out by *β*-ketoacyl-ACP synthase III (*mt*FabH) (Brown *et al.*, 2005; Choi *et al.*, 2000). This newly formed *β*-ketoacyl-ACP is reduced by a *β*-ketoacyl-ACP reductase (MabA) (Banerjee *et al.*, 1998) to form a *β*-hydroxyl-acyl-ACP intermediate. The product is then dehydrated by *β*-hydroxyacyl-ACP dehydratase (designated FabA and FabZ in *Escherichia coli*), followed by further reduction with the enoyl-ACP reductase, InhA, to complete the FAS-II cycle (Banerjee *et al.*, 1994; Kikuchi & Kusaka, 1984). Subsequent FAS-II cycles are initiated by the acyl-ACP-primed *β*-ketoacyl-ACP synthases KasA and KasB, respectively (Kremer *et al.*, 2000; Mdluli *et al.*, 1998; Schaeffer *et al.*, 2001), to afford a meromycolic acid (C_56_), which is then condensed with a C_26_ fatty acid (Gande *et al.*, 2004; Portevin *et al.*, 2005; Takayama *et al.*, 2005). The oxomycolic acid intermediate is then reduced to form the mature mycolic acid (Lea-Smith *et al.*, 2007).

**Fig. 1. f1:**
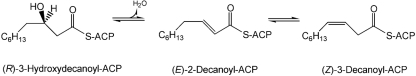
Dehydration and isomerization of 3-hydroxydecanoyl-ACP by *E. coli* FabA.

The dehydratase enzymes FabZ and FabA have been extensively studied in both *E. coli* and *Plasmodium falciparum* (Leesong *et al.*, 1996; Sharma *et al.*, 2003). Both FabZ and FabA catalyse the dehydration of the *β*-hydroxylacyl-ACP to a *trans*-2-enoyl ACP in the third step of fatty acid elongation. In addition to performing the dehydration step, FabA has the ability to isomerize *trans*-2- to *cis*-3-decanoyl-ACP (Fig. 1), as an essential step in the formation of unsaturated fatty acids in *E. coli* (Kass & Bloch, 1967; Kass *et al.*, 1967). The pivotal role played by FabZ and FabA makes them good potential drug targets against *M. tuberculosis*. The identification of the key FAS-II dehydration step in mycobacteria has remained an enigma until very recently (Sacco *et al.*, 2007).

In an attempt to establish whether Rv0636 represented the potential dehydratase candidate, overexpression studies were performed in *M. bovis* BCG against a series of flavonoid inhibitors known to target FabZ (Brown *et al.*, 2007b). Of the five flavonoids tested, four were found to be active against *M. bovis* BCG with MICs ranging from 150 to 220 μM, the most potent being butein. The activity of the flavonoids against the hypothesized gene product Rv0636 indicated that the overexpression in *M. bovis* BCG conferred resistance to butein and isoliquirtigenin (Brown *et al.*, 2007b). The data suggested that the flavonoids are inhibitors of mycobacterial FAS-II and in particular Rv0636, reiterating the potential candidacy of this gene product as the dehydratase enzyme of the FAS-II in *M. tuberculosis*.

Sacco *et al.* (2007) had independently demonstrated that the Rv0635–Rv0637 operon encoded dehydratase activity. The recombinant expression of the candidate protein cluster, Rv0635-Rv0636-Rv0637, led to the formation of two heterodimers, Rv0635-Rv0636 (HadAB) and Rv0636-Rv0637 (HadBC), which were shown to also occur in *Mycobacterium smegmatis* (Sacco *et al.*, 2007). Both heterodimers exhibited the enzymic properties expected for mycobacterial FAS-II dehydratases, including a marked specificity for both long-chain (>C_12_) and ACP-linked substrates (Sacco *et al.*, 2007). Furthermore, the authors of this study were able to show the function of Rv0636 or HadAB/ HadBC as a *β*-hydroxyacyl dehydratase when coupled with MabA and InhA enzymes from *M. tuberculosis* FAS-II.

Further research into potential dehydratase inhibitors has yielded the identification of NAS-21 and NAS-91, which have been shown to target *β*-hydroxyacyl-ACP dehydratase FabZ of *P. falciparum* (Sharma *et al.*, 2003). A decrease in the rate of enzyme activity was observed in the presence of both NAS-21 and NAS-91 using spectrometric and HPLC methods. The authors of that study also showed that the incorporation of [2-^14^C]malonyl-CoA into fatty acids in cell-free extracts of *P. falciparum* was inhibited to different extents by NAS-21 and NAS-91. The incorporation of [1, 2-^14^C]acetic acid into fatty acids was reduced by 26 and 46 %, respectively, in the presence of 10 μM NAS-21 and NAS-91. To investigate the potential anti-mycobacterial therapeutic activity of NAS-21 and NAS-91, we synthesized a library of these FabZ inhibitors. Using a similar strategy to that previously presented (Brown *et al.*, 2007b), we evaluated the analogues for their whole-cell activity against *M. bovis* BCG and an Rv0636-overexpressing *M. bovis* BCG strain, and and their *in vitro* activity against FAS-I and FAS-II in cell-free assays using *M. smegmatis* extracts.

## METHODS

### Synthesis of NAS-21 analogues.

**Scheme 1. f4:**
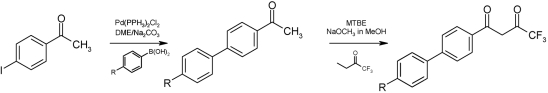
Method for production of NAS-21 analogues.

A series of NAS-21 analogues were developed using a previously described method (Sharma *et al.*, 2003) (Scheme 1). In brief, acetophenone derivatives were condensed with ethyl trifluoroacetate in the presence of 25 % NaOMe (in MeOH) and methyl-*tert*-butyl ether. Diversity was introduced into these reactions using a variety of commercially available acetophenone derivatives, yielding analogues **1**–**6** (Table 1). Biphenyl analogues **7**–**9** were developed via Suzuki coupling of 4-iodoacetophenone with aryl boronic acid derivatives. The product was then subjected to treatment with ethyl trifluoroacetate, 25 % NaOMe in MeOH and MTBE (Scheme 1) to yield the desired analogues **7**–**9**. An example of the Suzuki coupling reaction for analogue **7** is as follows. 4-Iodoacetophenone (100 mg, 0.292 mmol, 1 eq.), ethylene glycol dimethyl ether (3 ml), aqueous Na_2_CO_3_ (0.5 ml, 1 M) and 4-fluorophenylboronic acid (48.97 mg, 0.37 mmol, 1.2 eq.) in a round-bottom flask was degassed for 10 min. Bis(triphenylphosphine) palladium chloride (8 mg, 7×10^−3^, 5 mol%) was then added and the mixture was heated under reflux for 6 h. The mixture was partitioned between water (10 ml) and ethyl acetate (10 ml) and separated. The aqueous layer was acidified to pH 2 with dilute hydrochloric acid (2 M) and the product was extracted with ethyl acetate (2×10 ml). The organic layers were combined, washed with saturated brine (3×10 ml), dried and reduced to give the crude product. Purification was achieved via two separate silica gel columns. The first column used chloroform in methanol (95 : 5, v/v) as eluant and the second column used ethyl acetate in petroleum ether (35 : 65). The title analogue gave a white solid in 78 % yield (62 mg). ^1^H NMR (CDCl_3_, 300 MHz) *δ*_H_: 2.60 (s, 3H, CH_3_, H-14), 7.05 (d, 2H, H-5, H-7, *J=*8.5 Hz), 7.50 (d, 2H, H-4, H-8, *J=*8.5 Hz), 7.60 (d, 2H, H-3, H-9, *J=*8.0 Hz), 7.95 (d, 2H, H-2, H-10, *J=*8.0 Hz). ^13^C NMR (CDCl_3_, 75 MHz) *δ*_C_: 23.8 (C-14), 116.0, 127.4, 129.8 (C-2, 3, 4, 5, 7, 8, 9, 10), 132.0 (C-12), 136.7 (C-1), 142.0 (C-11), 168.4 (C-6), 195.6 (C-13); *m/z* (EI) 214.2 [M^+^] (100 %); HRMS calculated for C_14_H_11_FO [M^+^] 214.2319 found 214.2327.

### Synthesis of NAS-91 analogues.

**Scheme 2. f5:**
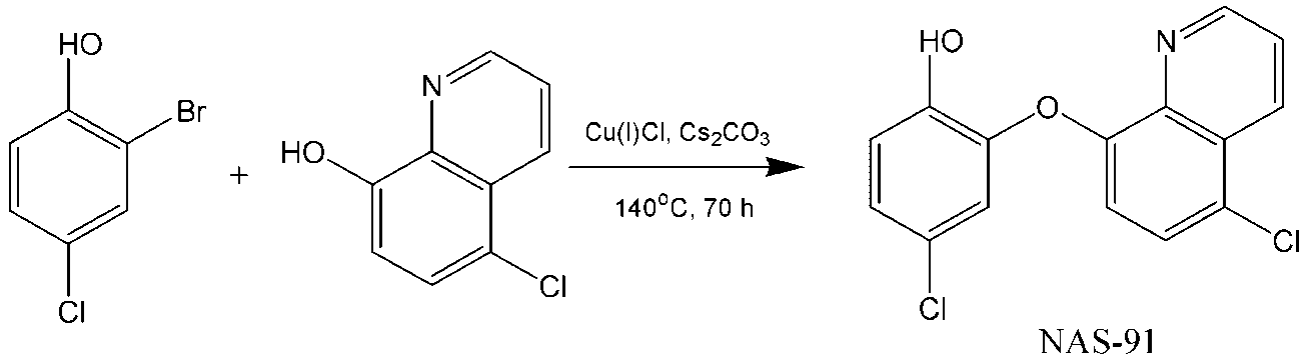
Method for production of NAS-91 analogues.

**Scheme 3. f6:**
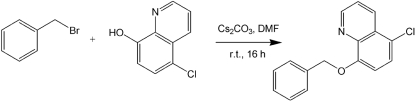
Method for adding a linker arm to 5-chloro-8-hydroxyquinolone.

NAS-91 was synthesized as described by Sharma *et al.* (2003). The reaction involves the coupling of 2-bromo-4-chlorophenol with 5-chloro-8-hydroxyquinolone, using caesium carbonate, copper (I) chloride (0.5 eq.) and *N*-methylpyrrolidinone as the solvent (Scheme 2) (Ullmann & Sponagel, 1905). An alternative method was developed to synthesize the remaining NAS-91 analogues in Table 2. A linker arm was introduced into 5-chloro-8-hydroxyquinolone by reacting it with benzyl bromide derivatives under basic conditions (Scheme 3). Diversity was introduced into this library by utilizing a variety of commercially available benzyl bromide derivatives. This method was employed to generate a library of seven novel NAS-91 analogues (**10**–**16**) (Table 2), which contain a methylene linker arm connected to the oxygen of 5-chloro-8-hydroxyquinolone. As an example, analogue **10** was synthesized as follows. 5-Chloro-8-hydroxyquinolone (500 mg, 2.78 mmol, 1 eq.) was dissolved in 5 ml dimethylformamide. To this was added caesium carbonate (452 mg, 1.39 mmol, 0.5 eq.). After 20 min of mixing at room temperature, benzyl bromide (0.37 ml, 3.06 mmol, 1.1 eq.) was added dropwise and the reaction was stirred at room temperature overnight. The reaction mixture was quenched with water. The organic layer was extracted with ethyl acetate, washed with water and brine, dried and reduced *in vacuo* to yield the crude product. The title analogue **10** was recrystallized to give a white solid in 85 % yield (635 mg). ^1^H NMR (CDCl_3_, 300 MHz) *δ*_H_: 5.35 (s, 2H, CH_2_, H-11), 6.85 (d, 1H, H-7, *J*=8.44 Hz), 7.15–7.45 (m, 4H, H-12, H-14, H-15, H-16), 7.48–7.50 (m, 3H, H-3, H-6, H-13), 8.40 (d, 1H, H-4, *J*=8.54 Hz), 8.90 (d, 1H, H-2, *J*=4.17 Hz). ^13^C NMR (CDCl_3_, 75 MHz) *δ*_C_: 68.9 (C-11), 107.8 (C-7), 120.4 (C-3), 124.3 (C-5), 124.5 (C-6), 125.2 (C-13, C-17), 126.1, 126.8, 127.2 (C-15, C-16, C-17), 130.8 (C-4), 134.5 (C-12), 138.1 (C-9), 147.8 (C-2), 153.2 (C-8); 3019.6m, 1638.3m, 1215.6s; *m/z* (EI) 369.06 [M^+^] (30 %), 91.00 [C_6_H_6_CH_2_^+^] (100 %); HRMS calculated for C_16_H_12_ClNO [M^+^] 269.0607 found 269.0603.

### Bacterial strains, growth conditions and MIC_99_ determination.

All reagents were of assay grade and purchased from Sigma-Aldrich. Overexpression of pVV16-Rv0636 (Brown *et al.*, 2007b) was conducted in *M. bovis* BCG on Middlebrook 7H10 agar supplemented with oleic-albumin-dextrose-catalase (OADC) enrichment (BD and Company) and containing 25 μg kanamycin ml^−1^ and 50 μg hygromycin ml^−1^ (Kremer *et al.*, 1995). Liquid cultures of *M. bovis* BCG were grown at 37 °C in Sauton's medium containing 25 μg kanamycin ml^−1^ and 50 μg hygromycin ml^−1^. MIC_99_ values of NAS analogues against *M. bovis* BCG/pVV16 and *M. bovis* BCG/pVV16-Rv0636 were determined by Alamar Blue as described previously using the manufacturer's protocol (Celltiter-Blue; Promega) followed by MIC_99_ calculations over the concentration range 0–200 μg ml^−1^ (Franzblau *et al.*, 1998).

### Determination of the whole-cell effects of NAS analogues on fatty acid and mycolic acid synthesis.

*M. bovis* BCG cultures were grown to OD_600_ 0.4 in the presence of 0.25 % Tween 80. The NAS analogues were added at various concentrations followed by incubation at 37 °C for 8 h and then 1 μCi (37 kBq) ml^−1^ [1,2-^14^C]acetate (50–62 mCi mmol^−1^, GE Healthcare, Amersham Bioscience) was added to the cultures, followed by further incubation at 37 °C for 16 h. The ^14^C-labelled cells were harvested by centrifugation at 2000 ***g*** followed by washing with PBS. The ^14^C-labelled control and NAS-treated cells were then subjected to alkaline hydrolysis using 5 % aqueous tetrabutylammonium hydroxide at 100 °C overnight, followed by the addition of 4 ml CH_2_Cl_2_, 500 μl CH_3_I and 2 ml water, followed by mixing for 30 min. The upper aqueous phase was discarded following centrifugation and the lower organic phase washed three times with water and evaporated to dryness. The resulting fatty acid methyl esters (FAMEs) and mycolic acid methyl esters (MAMEs) were redissolved in diethyl ether, and the supernatant was again removed after centrifugation and evaporated to dryness and redissolved in 200 μl CH_2_Cl_2_. An equivalent aliquot (20 μl) or equal counts (50 000 c.p.m.) of the resulting solution of FAMEs and MAMEs was subjected to TLC using silica gel plates (5735 silica gel 60F_254_; Merck), developed in petroleum ether/acetone (95 : 5). Autoradiograms were produced by overnight exposure to Kodak X-Omat AR film to reveal ^14^C-labelled FAMEs and MAMEs. Alternatively, free lipids were extracted from the ^14^C-labelled cells and crude lipids examined by TLC for PGL and phospholipid synthesis using the procedures of Dobson *et al.* (1985).

### Preparation of cytosolic fractions, and FAS-I and FAS-II assays.

Cytosolic extracts, enriched for FAS-I and FAS-II using ammonium sulphate precipitation, of *M. smegmatis* mc^2^155/pVV16 and *M. smegmatis* mc^2^155/pVV16-Rv0636 (approx. 10 g) were prepared as described previously (Kremer *et al.*, 2002a). The final extract containing the FAS-I and FAS-II activities was dissolved in 5 ml 50 mM MOPS pH 7.9, 5 mM *β*-mercaptoethanol, 10 mM MgCl_2_. Protein concentrations were determined using the BCA protein assay reagent kit (Pierce). FAS-I and FAS-II assays were conducted as previously described using the 40–80 % ammonium sulfate fraction (Kremer *et al.*, 2002b; Slayden *et al.*, 1996).

## RESULTS AND DISCUSSION

### Biological evaluation of NAS-21 analogues

NAS-21 has been shown to target the *β*-hydroxylacyl-ACP dehydratase FabZ of *P. falciparum* (Sharma *et al.*, 2003). The mycobacterial FabZ (Rv0635–637) is a potentially attractive target for such an inhibitor; therefore we sought to synthesize and evaluate NAS-21 analogues against *M. bovis* BCG, which possesses a similar drug profile to *M. tuberculosis* in terms of sensitivity (Larsen *et al.*, 2002; Vilcheze *et al.*, 2005). The results of the whole-cell analysis of NAS-21 analogues against *M. bovis* BCG pVV16 and *M. bovis* BCG/pVV16-Rv0636, which overexpresses Rv0636, are shown in Table 1. It is clearly evident that the COCH_2_COCF_3_ group plays a central role in the activity of NAS-21 analogues, because the simple conversion of this group to the COCH_3_ functionality resulted in the inactivation of the compounds (MIC_99_>250 μg ml^−1^) (data not shown). Two possible explanations for this observed decrease in activity are (i) the di-keto nature of the analogue may mimic the *β*-keto substrate utilized in FAS-II and may act as a competitive inhibitor or (ii) the presence of the CF_3_ group may stabilize the formation of the keto–enol tautomer of these analogues, which may increase the interaction of the drug with the enzyme's active site. Extension in the *para*-position of the aromatic ring in NAS-21 resulted in analogues with a significant reduction in activity (Table 1). This is demonstrated by the effects of (i) the simple modification of the methyl group in **4** to an ethyl group in **5** and (ii) the introduction of a biphenyl group (**7**–**9**), thus indicating that modifications extending in this position are not well tolerated. Comparable whole-cell activity of the parent drug NAS-21 and analogues **1**, **2** and **4** was demonstrated against *M. bovis* BCG/pVV16. A significant decrease in whole-cell activity against the Rv0636-overexpressing *M. bovis* BCG strain was also observed, suggesting that the product of this gene may represent the cellular target for NAS-21 and analogues **1**, **2** and **4**.

### Effects of NAS-21 analogues on activity of FAS-I and FAS-II in cell-free extracts

To further evaluate the biological properties of NAS-21 analogues and to investigate their potential target, Rv0636, a series of *in vitro* FAS-I and FAS-II assays were performed on crude cell-free extracts of *M. smegmatis* as previously described by Slayden *et al.* (1996). The analysis was performed on extracts isolated from both *M. smegmatis*/pVV16 and *M. smegmatis*/pVV16-Rv0636. The activity of each analogue was measured by the incorporation of radiolabel into extractable lipids. Specific assays were utilized by using priming units in the form of two different fatty acyl-CoAs, either acetyl-CoA or palmitoyl-CoA, for FAS-I or FAS-II, respectively. In both cases [1, 2-^14^C]malonyl-CoA was utilized as the radiolabelled carbon donor. In the case of FAS-I, [1,2-^14^C]malonyl-CoA coupled with acetyl-CoA to form short-chain fatty acids. However, in the case of FAS-II, [1,2-^14^C]malonyl-CoA is transacylated by *mt*FabD to form [1,2-^14^C]malonyl-AcpM, which is subsequently used for the initiation of FAS-II by *mt*FabH (Choi *et al.*, 2000). AcpM supplementation in the FAS-II assays drives the reaction towards the production of [1,2-^14^C]malonyl-AcpM. Inhibition (IC_50_) values were determined by varying the concentrations of the drug and by measuring the incorporation of radioactivity into extractable lipids. The results of the crude cell-free extract assay revealed that none of the analogues synthesized inhibited FAS-I (data not shown). Encouragingly, an increased activity was observed for most analogues against FAS-II (Table 1). Analogues **1**–**6** gave good *in vitro* activity against the cell-free *M. smegmatis* pVV16 extracts of FAS-II. In particular **3**, **4** and **5** gave IC_50_ values of 35, 28 and 19 μg ml^−1^, respectively, against the *M. smegmatis*/pVV16 FAS-II extract. An increase in resistance was also observed for *M. smegmati*s/pVV16-Rv0636, further suggesting Rv0636 to be a potential target of the analogues. Interestingly, the whole-cell analysis of analogues **3** and **5** gave very poor activities (Table 1), indicating that these modifications affect the permeability of the drug across the cell wall or that they are modified prior to reaching their target. It was also interesting that analogues **1** and **2**, which gave the most pronounced effects against whole cells of *M. bovis* BCG/pVV16 and *M. bovis* BCG/pVV16-Rv0636, did not give the same marked response compared to **4** and **5** in relation to FAS-II inhibition with the same strains (Table 1). Analogues **7**–**9** were inactive against whole cells; however, moderate activity was observed in FAS-II assays (Table 1), implying that cell permeability may be a contributing factor towards the lack of whole-cell activity of these biphenyl-containing analogues.

### Biological evaluation of NAS-91 analogues

NAS-91 showed poor whole-cell activity against both *M. bovis* BCG/pVV16 and *M. bovis* BCG/pVV16-Rv0636 and no inhibition was observed even at high concentrations (>250 μg ml^−1^) (Table 2). The observed poor inhibition of *M. bovis* BCG growth was surprising since Gratraud *et al.* (2008) recently reported an MIC value of 25 μg ml^−1^ against *M. bovis* BCG for NAS-91, although the MIC values reported for NAS-21 (50 μg ml^−1^) by Gratraud *et al.* (2008) are similar to the values (63 μg ml^−1^) reported in this study. A key feature of note in the studies by Gratraud *et al.* (2008) was that the MIC values were determined on Middlebrook 7H11 agar plates by visualizing plaques following serial dilution. In contrast, in this present study, MIC values for NAS-91 (as well as NAS-21) were determined using the more established and sensitive Alamar Blue method (Franzblau *et al.*, 1998) in Sauton's liquid medium. It is clear that MIC values for NAS-91 in particular are different on liquid and solid media. This is not totally surprising since similar observations have been reported for drug inhibition of mycobacterial strains. For instance, *M. smegmatis* is sensitive to econazole and clotrimazole on LB solid agar plates, with MIC values of 2 and 0.5 μg ml^−1^, respectively (Burguiere *et al.*, 2005). However, when *M. smegmatis* is cultured in Sauton's liquid medium, the MIC values are higher than those determined on agar plates, with econazole at 20 μg ml^−1^ (10-fold higher) and clotrimazole at 15 μg ml^−1^ (30-fold higher), respectively. Interestingly, it is clear that a concentration of 100 μg ml^−1^ of NAS-91 in liquid media is only partially inhibiting mycolate synthesis (50 %) in the Gratraud *et al.* (2008) study, which is at four times the MIC value on solid media. This is further evidence for the MIC value for NAS-91 being different on solid and liquid media.

Analogues **13**–**16** demonstrated significantly improved whole-cell activity in comparison to NAS-91. The simple introduction of a methyl modification in analogue **15** resulted in the most improved whole-cell activity, with an MIC_99_ value of 18 μg ml^−1^ against *M. bovis* BCG/pVV16. Encouragingly, resistance was shown against analogue **15** when *M. bovis* BCG/pVV16-Rv0636 was used, with an increase in MIC_99_ to 100 μg ml^−1^. Structurally, analogues **13** and **14** indicate that there is more scope to extend the modification in the *ortho*-position by two or more carbons. Analogues **10** and **11** were primarily developed to assess the feasibility of introducing a linker arm into the analogues whilst changing the functionalities on the aromatic ring. As indicated in Table 2, the low biological activity of these analogues was comparable to that of NAS-91. Initially it was felt that the linker arm might have compromised activity by reorientating the analogue within the active site, thus affecting its interactions with the target. However, as observed with analogues **13**–**16**, modifications in the *ortho*-position of the aromatic ring greatly increase the potency of this analogue, suggesting it is the nature of the modification on **10** and **11** which has compromised their whole-cell activity. From the activities observed with analogues **13–16** it is evident that the hydroxyl group of the secondary aromatic functionality does not play an important part in the protein–drug interaction, as activity was still observed in these analogues. Finally, the introduction of a second aromatic group in the *para*-position (**12**) compromised the whole-cell activity against both *M. bovis* BCG/pVV16 and *M. bovis* BCG/pVV16-Rv0636. This initial study suggests that there is limited scope to further extend in the *para*-position with a second aromatic ring; however, this requires verification by formulating a more comprehensive library.

### Effects of NAS-91 analogues on activity of FAS-I and FAS-II in cell-free extracts

To further evaluate the activities of the NAS-91 analogues, a series of *in vitro* FAS-I and FAS-II assays were performed on crude cell-free extracts of *M. smegmatis*. As with the NAS-21 analogues, the crude *M. smegmatis* cell-free FAS-I assays revealed that none of the analogues inhibited FAS-I (data not shown). Analogues **10** and **12** demonstrated similar effects to NAS-91 against FAS-II activity in *M. smegmatis* cell-free extracts (Table 2). Encouragingly, analogues **11** and **13**–**15** gave a marked increase in *in vitro* activity against FAS-II, and extracts from *M. smegmatis*/pVV16-Rv0636 FAS-II extract showed resistance to these analogues. Analogues **13**–**15** also showed good whole-cell activity against whole-cell *M. bovis* BCG/pVV16, providing further evidence that these analogues would form a good basis to generate a secondary library of NAS-91 analogues.

### Effects of NAS-21 and NAS-91 analogues on FAME and MAME synthesis

**Fig. 2. f2:**
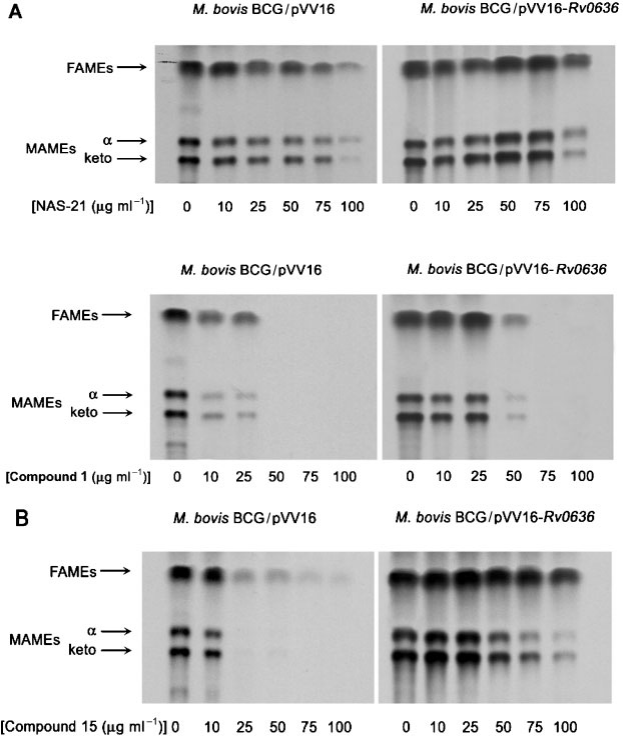
TLC-autoradiography of *M. bovis* BCG FAMEs and MAMEs after NAS-21 and NAS-91 analogue treatment. (A) NAS-21 analogue 1 (0–100 μg ml^−1^) and (B) NAS-91 analogue 15 (0–100 μg ml^−1^) were titrated into the *M. bovis* BCG/pVV16 cultures at an OD_600_ of 0.4 prior to labelling with 1 μCi (37 kBq) [1,2-^14^C]acetate ml^−1^ for 8 h. [^14^C]FAMEs and [^14^C]MAMEs were extracted and resolved by TLC. An equivalent aliquot (20 μl) of the resulting solution of FAMEs and MAMEs was subjected to TLC using silica gel plates (5735 silica gel 60F_254_; Merck), developed in petroleum ether/acetone (95 : 5, v/v). Autoradiograms were produced by overnight exposure to Kodak X-Omat AR film to reveal ^14^C-labelled FAMEs and MAMEs.

**Fig. 3. f3:**
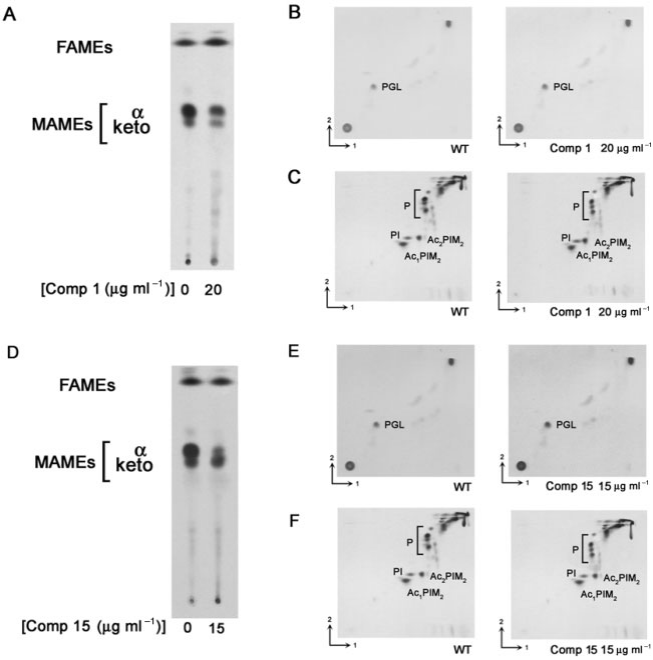
TLC-autoradiography of *M. bovis* BCG lipids after NAS-21 and NAS-91 analogue treatment. (A, D) Analysis of FAMEs and MAMEs following treatment with NAS-21 analogue 1 (20 μg ml^−1^) and NAS-91 analogue 15 (15 μg ml^−1^) and resolved by TLC using equal counts (50 000 c.p.m.) as described in Methods. Lipid extractions were performed as described by Dobson *et al.* (1985) and a 50 000 c.p.m. aliquot analysed using silica gel plates (5735 silica gel 60F_254_; Merck). (B, E) Phenolic glycolipids (PGL) were identified by 2D TLC [direction 1, chloroform/methanol (94 : 4, v/v); direction 2, toluene/acetone (80 : 20, v/v)]. (C, F) Phospholipids (P), phosphatidylinositol (PI), acyl-phosphatidylinositol dimannoside (Ac_1_PIM_2_) and diacyl-phosphatidylinositol dimannoside (Ac_2_PIM_2_) were identified by 2D TLC [direction 1, chloroform/methanol/water (60 : 30 : 6, by vol.); direction 2, chloroform/acetic acid/methanol/water (40 : 25 : 3 : 6, by vol.)]. Autoradiograms were produced by overnight exposure to Kodak X-Omat AR film to reveal ^14^C-labelled FAMEs, MAMEs and lipids.

*M. bovis* BCG/pVV16 was grown in the presence of the NAS analogues at various concentrations, followed by [1,2-^14^C]acetate labelling and analysis by TLC separation of FAMEs and MAMEs. An example of the results, for analogues **1** and **15**, is shown in Fig. 2. There was a decrease in the incorporation of radioactivity into FAMEs and MAMEs in the presence of NAS-21, analogues **1** and **15**. Since analogues **1** and **15** were shown not to inhibit FAS-I (data not shown), the experiment was repeated; equal counts were loaded and the TLC profiles of FAMEs and MAMEs reanalysed (Fig. 3A, D[Fig f3]). It is clear from this analysis that analogues **1** and **15** only inhibit the synthesis of *α*- and keto-MAMEs and not that of FAMEs (Fig. 3), consistent with the earlier *in vitro* data (Tables 1 and 2). As an additional control the synthesis of cell envelope lipids was also examined (Fig. 3). Analogues **1** and **15** again do not inhibit general fatty acid synthesis as the synthesis of PGL (Fig. 3B, E[Fig f3]) and phospholipids (Fig. 3C, F[Fig f3]) remains unaffected. Resistance was also observed upon the overexpression of pVV16-Rv0636, supporting the earlier MIC_99_ and *in vitro* studies and thereby strengthening the evidence that these analogues target Rv0636 (Fig. 2). Similar results were observed with the other active analogues (**2**, **4**, **13**, **14** and **16**).

### Concluding remarks

In conclusion, no activity was observed against FAS-I for either NAS-21 or NAS-91. In general, all the analogues showed *in vitro* activity against FAS-II extracts, and the Rv0636-overexpressing strain carrying pVV16-Rv0636 showed a marked increase in resistance. Whole-cell FAME and MAME analysis for most analogues demonstrated a decrease in both mycolic acid and fatty acid biosynthesis. Interestingly, this effect of the analogues was also reduced in *M. bovis* BCG/pVV16-Rv0636, thus further implicating Rv0636 as the target for these FabZ dehydratase inhibitors. The present study extends the initial findings of Gratraud *et al.* (2008), who did not perform FAS-I and FAS-II *in vitro* enzyme studies, using NAS-21 and NAS-91 to examine mycolate inhibition directly. Although the study of Gratraud *et al.* (2008) demonstrated that NAS-21 and NAS-91 also inhibited oleate biosynthesis it is clear that this represents a secondary target since it is non-essential, in contrast to Rv0636, which has been shown to be essential (Brown *et al.*, 2007a). In comparison to the FAS-II flavonoid inhibitors (Brown *et al.*, 2007b), our NAS-21 and NAS-91 analogues demonstrated a marked enhancement in activity; in some cases an eightfold increase is observed. Therefore NAS-21 and NAS-91 analogues represent good candidates for further development of drugs targeting the mycobacterial FAS-II dehydratase. However, to fully establish the potential therapeutic properties of NAS-21 and NAS-91, their *in vitro* activity against the heterodimers Rv0635-Rv0636 (HadAB) and Rv0636-Rv0637 (HadBC) must be also evaluated. The recent development of an *in vitro* assay for the FAS-II dehydratase activity (Sacco *et al.*, 2007) will help us to better understand the inhibitory activity of these compounds.

## Figures and Tables

**Table 1. t1:**
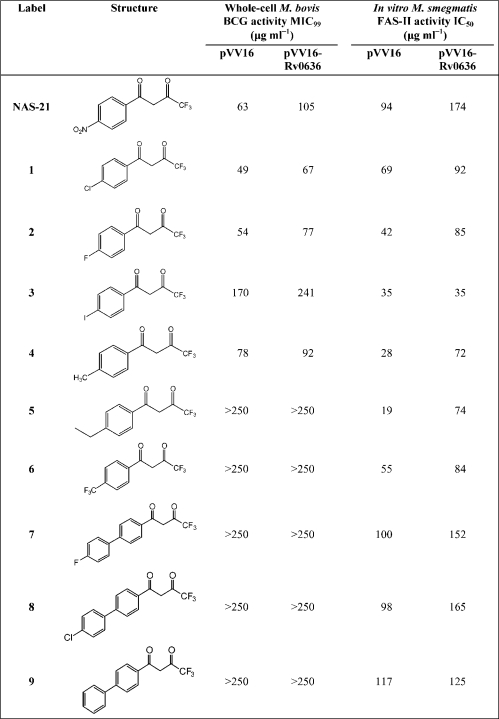
Structures of NAS-21 analogues, whole-cell inhibitory activity against *M. bovis* BCG and *in vitro* inhibition of *M. smegmatis* FAS-II activity

**Table 2. t2:**
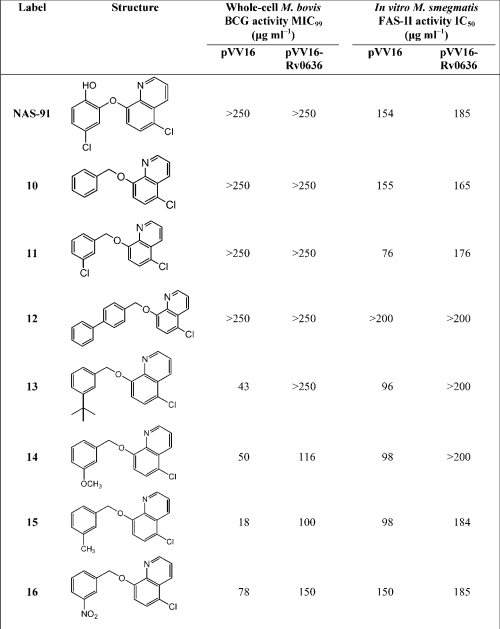
Structures of NAS-91 analogues, whole-cell inhibitory activity against *M. bovis* BCG and *in vitro* inhibition of *M. smegmatis* FAS-II activity

## Abbreviations

- ACP, acyl carrier protein
- FAMEs/MAMEs, fatty/mycolic acid methyl esters
- FAS, fatty acid synthase
- MeOH, methanol
- MTBE, methyl *tert*-butyl ether
- NaOMe, sodium methoxide
- TB, tuberculosis
